# Expert consensus document on automated diagnosis of the electrocardiogram: The task force on automated diagnosis of the electrocardiogram in Japan: Part 2: Current status of inappropriate automated diagnosis is widely used electrocardiographs in Japan

**DOI:** 10.1002/joa3.12646

**Published:** 2021-10-19

**Authors:** Takao Katoh, Masaaki Yashima, Naohiko Takahashi, Eiichi Watanabe, Takanori Ikeda, Yuji Kasamaki, Naokata Sumitomo, Norihiro Ueda, Hiroshi Morita, Masayasu Hiraoka

**Affiliations:** ^1^ Clinic of Tobu Railway Co. Ltd. Sumida‐ku Japan; ^2^ Nippon Medical School Department of Cardiology Bunkyo‐ku Japan; ^3^ Oita University Department of Cardiology and Clinical Examination Yufu Japan; ^4^ Fujita Health University Bantane Hospital Toyoake Japan; ^5^ Toho University Medical Center Omori Hospital Tokyo Japan; ^6^ Kanazawa Medical University Himi Medical Center Himi Japan; ^7^ Saitama Medical University International Medical Center Saitama Japan; ^8^ Nagoya City University Department of Medical Education Nagoya Japan; ^9^ Graduate School of Medicine Dentistry and Pharmaceutical Sciences Okayama University Okayama Japan; ^10^ Tokyo Medical & Dental University Bunkyo‐ku Japan

**Keywords:** automated diagnosis, ECG

## Abstract

It is important to objectively grasp the current status of automated electrocardiogram (ECG) diagnosis. This report aimed to analyze and evaluate ECG records that our members have encountered as an inappropriate diagnosis in real‐world clinical practices.

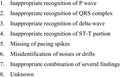

## 1 BACKGROUND

The first attempt at automatic interpretation of the electrocardiogram (ECG) using a computer was reported by Pipberger et al^1^ in1960. This was followed by Okajima et al,^2^ Kimura et al,^3^ and Matsuo et al^4^ in the 1960s using individual computer systems from Japan. Although the diagnostic accuracy has gradually improved over the past half‐century since then, it is still unsatisfactory for the expert electrocardiologist.^5^, ^6^, ^7^, ^8^

At present, electrocardiographic examination is widely used in cardiovascular medicine and other clinical fields including pre‐operative examinations and health checks for the general population. Thus the results of automated diagnosis obtained by a computer‐equipped electrocardiograph have been widely applied in clinical medicine and preventive medicine. As a result of its wide range of utility and application, the results of automated ECG diagnosis are shared and utilized in medical practice in various situations by cardiologists, specialized medical staff, practitioners in other fields, and paramedical personnel. Therefore, while automated ECG diagnosis requires high accuracy, there are in fact many problems and issues still to be resolved.^9^ Over‐reading of ECG by expert physicians is essential.^10^ Measures to improve the diagnostic accuracy of automated ECG diagnosis and to further enhance its clinical utility are of high importance.

In order to achieve this, it is necessary to further improve the accuracy of automatic ECG interpretation, such as by utilizing artificial intelligence (AI) with an appropriate method.

As a first step, it is important to objectively grasp the current status of automated ECG diagnosis.

Thus this report aimed to analyze and evaluate ECG records that our members have encountered as an inappropriate diagnosis in real‐world clinical practices. We suppose that there are no substantial differences between the automated ECG device employed in this study manufactured by Fukuda Denshi Co. Ltd. (Tokyo, Japan), Nihon Kohden Corporation (Tokyo, Japan), Suzukenn Co. Ltd. (Nagoya, Japan) and those widely used in other countries in the automatic measurement algorithm for P, Q, R, S, T waves, and in the criteria for ECG diagnosis. However, a careful check of automatic measurement algorithm and diagnostic criteria in the individual device is required in extrapolating the present results to other devices used in other countries.

## 2 COLLECTION OF ECG THAT SHOWED AN INAPPROPRIATE AUTOMATED DIAGNOSIS

Xerox copies or electronic records of ECG data that have been judged to have an inappropriate automated diagnosis were collected. The collection period was approximately 6 months from spring to autumn 2018. The approval of the ethics committee or the equivalent organization for each member's facility was obtained in advance of the collection.

A total of 1524 de‐identified ECGs were collected from 12 task force members. As shown in Table 1, various types of ECG were included based on the characteristics of individual members' facilities or the intended collection of targeted data. An AC filter, a muscle filter, and a drift filter may be turned ON in many of the cases because they are sometimes applied automatically.

**TABLE 1:** Collection of ECGs with inappropriate automated diagnosis (in no particular order)

Members	No. of ECGs	Types of inappropriate diagnosis
A	272	Various (from out‐patient clinic of cardiology department in the university hospital)
B	14	Various arrhythmias and waveform abnormalities
C	2	Prolonged QT intervals
D	8	AV block and pacemaker ECGs
E	14	Various arrhythmias
F	10	WPW syndrome
G	2	WPW syndrome
H	30	Atrial fibrillation, WPW syndrome, P wave abnormalities
I	13	AV block, pacemaker ECGs, atrial fibrillation
J	31	Atrial fibrillation, old myocardial infarction
K	20	Atrial fibrillation, old myocardial infarction
L	1108	Various (from successive 50 000 cases of medical check‐up)
Total	1524	Various arrhythmias and waveform abnormalities

Abbreviations: AV, atrio‐ventricular; ECG, electrocardiogram; WPW, Wolf–Parkinson–White.

## 3 TYPES OF INAPPROPRIATE DIAGNOSIS

The 1524 collected ECG records were classified into the following six categories of inappropriate diagnosis.

### 3.1 Pattern 1: Over‐diagnosis

When developing computer algorithms for automated ECG interpretation, it is possible that diagnostic criteria are sometimes a little broad because of the concern that a lack of important diagnosis may cause serious clinical problems. For example, ST level elevation because of early repolarization in young people may be a normal variant, but there are many cases where “acute myocardial infarction” is displayed as a result of the automated diagnosis. In cases where there is poor R wave progression in V1, V2, there are many examples where a diagnosis of “anterior or anteroseptal myocardial infarction” has been made because of misreading as a Q wave or QS pattern.

**FIGURE 1:**
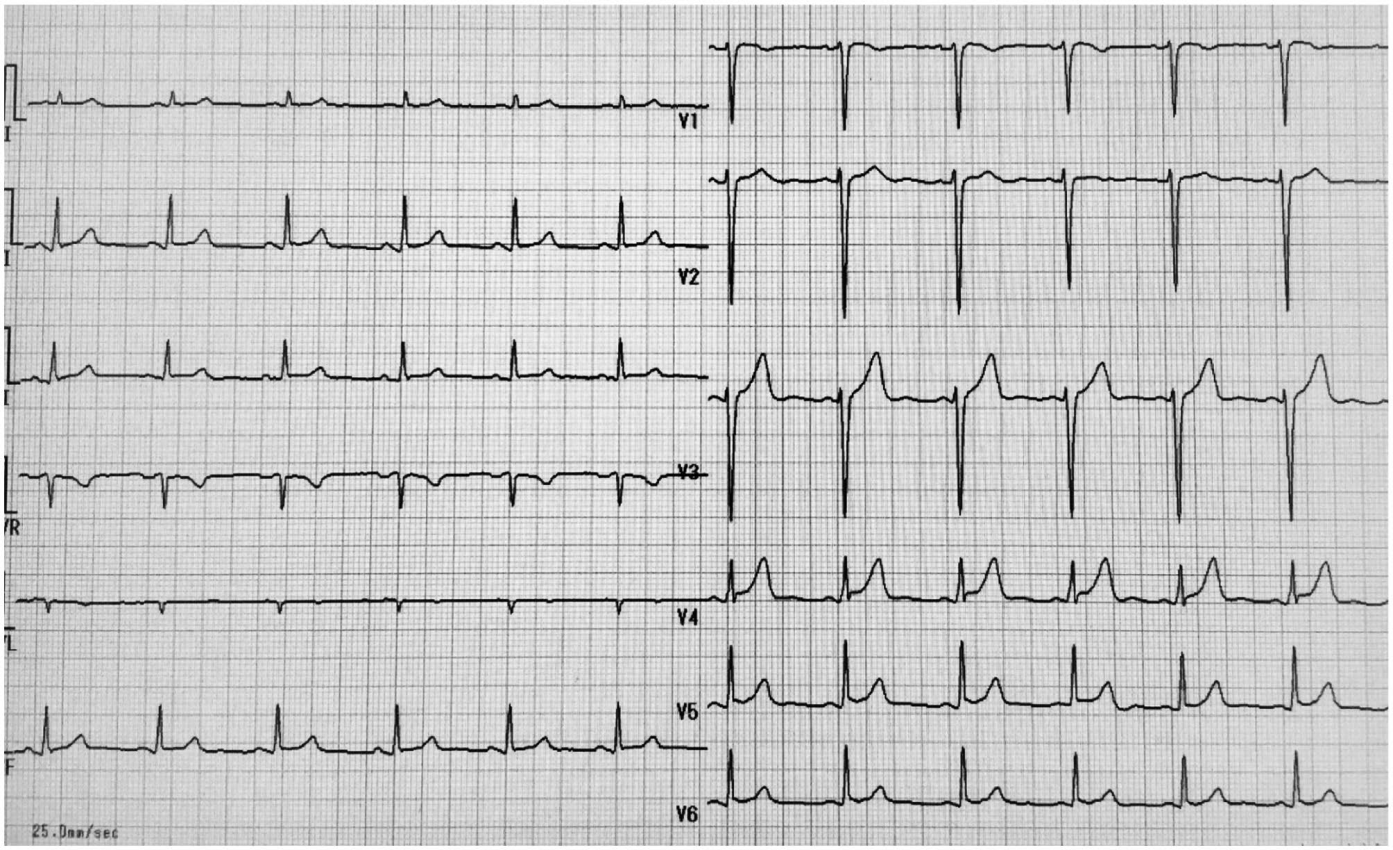
Case 1: 37 y‐old male. Recorded at employment medical check‐up. The diagnosis of “acute myocardial infarction, anterior wall” was made by automated interpretation at that time. He had no symptoms and was born healthy. It is assumed that relatively low R‐wave amplitude in leads V1‐V3 is associated with slight ST elevation in leads V4 and V5 probably because of early repolarization can lead to such an over‐diagnosis

A typical example is shown in Figure 1. Such types of over‐diagnosis as an inappropriate ECG interpretation are most frequently encountered, resulting in an unnecessary urgent consultation with an expert cardiologist.

### 3.2 Pattern 2: Under‐diagnosis

In view of the characteristics of the conventional algorithm of automated diagnosis described above, the occurrence of inappropriate diagnosis because of “under‐diagnosis” is infrequent compared with “over‐diagnosis.” However, an under‐diagnosis may cause serious problems in the clinical setting, if important abnormalities that require quick and appropriate action are missed.

**FIGURE 2:**
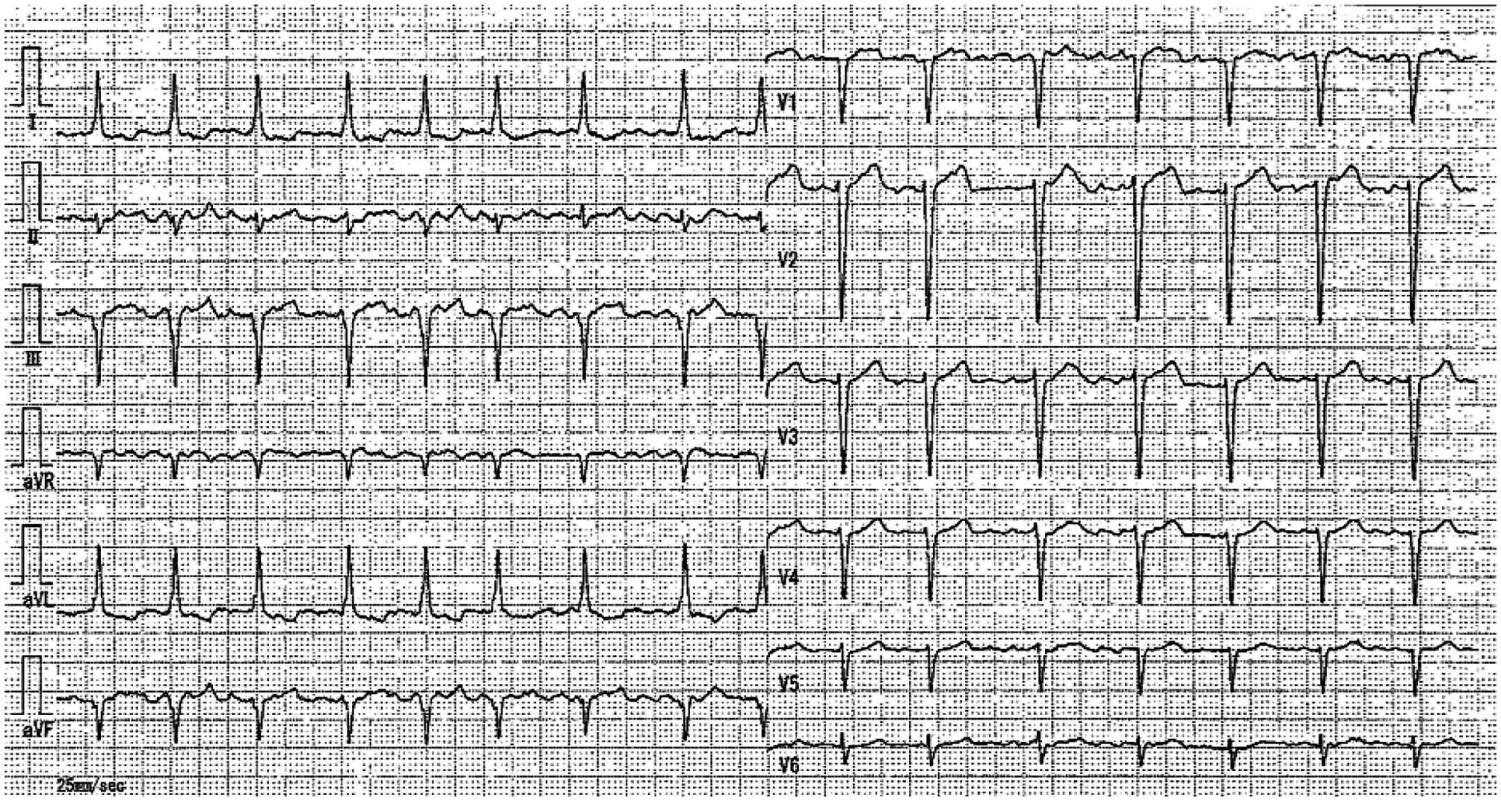
Case 2: 71 y‐old male. Recorded at medical checkup for local residents. The diagnosis of “first degree atrio‐ventricular block with sinus arrhythmia” was made by automated interpretation despite apparent electrocardiogram findings as atrial fibrillation. He was indicated a 1‐y follow‐up. It is assumed that overlooking of the f‐wave and non‐recognition of the absolute irregularity of the RR interval that are typical findings of atrial fibrillation can lead to such an inappropriate diagnosis

We have encountered several cases of atrial fibrillation that have been left untreated for many years because of diagnosis as a “normal sinus rhythm” because of low amplitude f‐wave and a relatively small irregularity in RR intervals. An example is shown in Figure 2.

### 3.3 Pattern 3: Inaccurate measurement

Essentially, automated diagnosis is based on results of precise measurements of P, Q, R, S, T wave including each onset points, offset points, PQ, QRS, and QT intervals. If the values of these parameters are inaccurate, the final diagnosis of the ECG becomes unreliable.

In particular, inaccurate measurements occur frequently at the onset of P wave and the offset of T wave because of their lenient waveforms, resulting in an inappropriate diagnosis such as “prolonged PQ (PR) interval” or “prolonged QT interval.”

In addition, it is still controversial as to which leads or how many beats should be used for automatic measurements. Primary discriminant function, probabilistic logics including Bayes' theory and bifurcation theory have been proposed as mathematical approaches for measurements of ECG parameters.^13^, ^14^, ^15^ It is speculated that the bifurcation theory is mainly used in the latest models of electrocardiograph. Since details are not disclosed by each manufacturer, it is difficult to clarify the reason for measurement errors when it happens.

### 3.4 Pattern 4: Inadequate algorism for diagnosis

The final diagnosis is made by combining the measured data of each waveform and interval. However, it is impossible to make an appropriate diagnosis if there is an inadequacy in the algorithm for each diagnostic process.

For example, although the diagnosis of “complete atrio‐ventricular (AV) block” is made by combining information including accurate detection of P waves and QRS complexes, accurate measurement of PP and RR intervals and the confirmation of AV dissociation showing no fixed relations between P waves and QRS complexes. Furthermore, marked bradycardia with a heart rate that is less than 50/min may appear with completely regular RR intervals. However, the diagnosis of complete AV block is often missed as AV dissociation findings are not recognized. Unfortunately, many detailed algorithms for automated diagnosis including complete AV block have not been disclosed.

**FIGURE 3:**
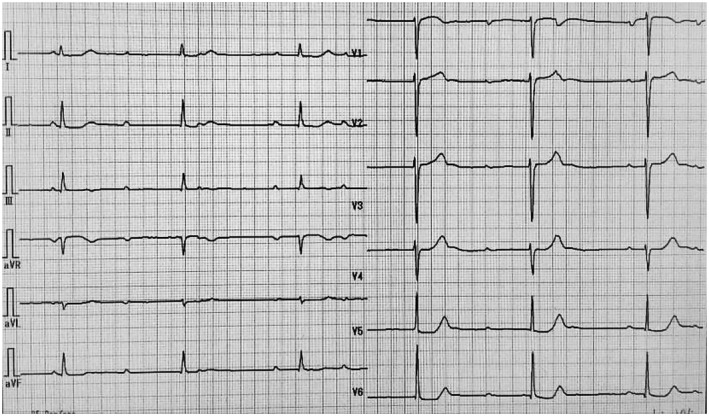
Case 3: 50 y‐old female. Recorded at her family physician. The patient consulted her family physician because she experienced dizziness with marked bradycardia. The automated electrocardiogram diagnosis indicated “first degree atrio‐ventricular (AV) block with bradycardia” despite apparent findings of complete AV block with extremely low ventricular rate of 35/min. She has been transferred to our hospital and was later implanted a permanent pacemaker. It is assumed that inappropriate recognition of P waves, inappropriate evaluation of PP and RR intervals and non‐recognition of typical findings for complete AV block might lead to such a problematic diagnosis. A drastic improvement of algorithms for diagnosis are urgently needed

An example is shown in Figure 3.

### 3.5 Pattern 5: Inappropriate notation of terms for diagnosis and ECG findings

A detailed suggestion for terminology used in the diagnosis or abnormal ECG findings has been published in the first report of consensus documents.^11^, ^12^ The terms of clinical diagnosis and mere ECG abnormalities were sometimes appear at the same level. In contrast, clinically insignificant ECG diagnosis have often been emphasized in surplus. Some notations frequently lead to misunderstanding for medical personnel who are not specialized in ECG.

For example, if a slight ST‐T abnormality was observed in leads V1 or V2, “Brugada syndrome suspected” may appear as a name in automated diagnosis. As “there is a risk of sudden death” for this condition, it may result in a request for immediate consultation with a specialist, even though it was unnecessary. Furthermore, a similar ECG finding may often be described as “RSR′ pattern,” “incomplete right bundle branch block,” etc Therefore, reliable algorithms are needed for discrimination, together with explanations on their clinical significance.

### 3.6 Pattern 6: Others

Electrocardiogram findings with low clinical significance or unclear definition including RSR′ pattern, S1S2S3 pattern, slight axis deviation, clockwise rotation or counterclockwise rotation, etc were sometimes emphasized at medical checkup, and “attention required” or “re‐examination required” were frequently instructed.

## 4 FREQUENCY OF INAPPROPRIATE AUTOMATED DIAGNOSIS IN CLINICAL PRACTICE

With regard to the frequency of inappropriate automated diagnosis in clinical practice, large‐scale reliable results have not been reported until now.

The reported frequency of inappropriate diagnosis differs depending on the type of institution to which the examiners belong to, that is, an institution for medical examination, a general hospital, a university hospital, or a special hospital for cardiovascular diseases. In fact, there was a huge bias by our members who submitted actual examples of inappropriate diagnosis as shown in Table 2.

**TABLE 2:** Primary causative factors of inappropriate diagnosis

Inappropriate recognition of P wave Inappropriate recognition of QRS complex Inappropriate recognition of delta wave Inappropriate recognition of ST‐T portion Missing of pacing spikes Misidentification of noises or drifts Inappropriate combination of several findings Unknown

Therefore, in order to estimate the approximate frequency of inappropriate automated diagnosis in the general population, consecutive 50 000 over‐read ECGs at medical checkup were investigated. Subsequently, 1108 cases were extracted as inappropriate diagnosis. The frequency of inappropriate automated diagnosis in the population was about 2.2% (1108 out of 50 000 cases), conversely, in nearly 98% of cases the automated diagnosis had been appropriate.

**FIGURE 4:**
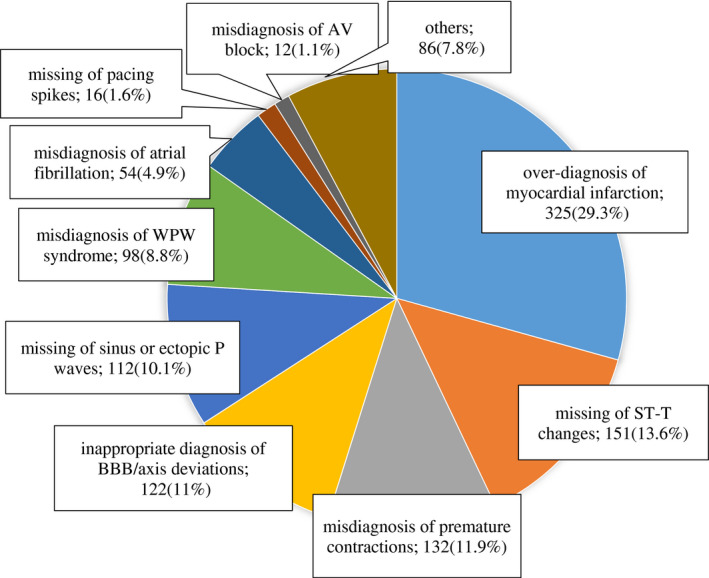
Frequency of inappropriate electrocardiogram diagnosis (1108 of 50 000 (2.2%) medical checkup cases). AV, atrio‐ventricular, BBB, bundle branch block, WPW, Wolf–Parkinson–White

Figure 4 shows the contents of inappropriate diagnosis of these 1108 cases in order of frequency.

The highest frequency was “over‐diagnosis of myocardial infarction” (29.3%). In contrast, “ST‐T changes” where ischemic heart disease could not be excluded were overlooked (13.6%). It is necessary to evaluate the ST‐T changes more accurately from the standpoint of medical examination.

Over‐diagnosis of left anterior hemiblock or left posterior hemiblock against slight axis deviations, and over‐diagnosis of ventricular premature contractions against supraventricular premature contractions with aberrant ventricular conduction also appeared frequently, although their clinical significance may not be important. These were thought to be because of inaccurate measurements and inadequate algorithms for the diagnosis. However, misdiagnosis of atrial fibrillation, overlooking of pacing spikes, and misdiagnosis of AV block in the medical examination must be avoided because it is likely to require a thorough checkup at a specialized cardiology institution. Fortunately, these did not appear frequently.

**FIGURE 5:**
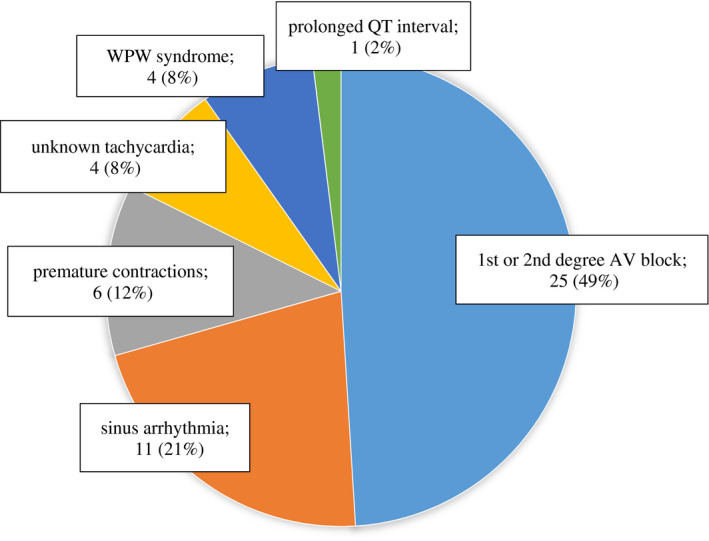
Results of automated diagnosis that overlooked atrial fibrillation (51 of 272 (18.8%) consecutive cases of inappropriate automated diagnosis). AV, atrio‐ventricular, WPW, Wolf–Parkinson–White

An accurate diagnosis of atrial fibrillation is increasingly important because of recent improvements in ablation therapy performance and the introduction of newly developed direct oral anticoagulants. Accordingly, a major revision of treatment guideline in Japan has been made in recent years.^16^ Inappropriate diagnosis of atrial fibrillation was seen in 54 out of 1108 patients (4.9%). Most of them were an over‐diagnosis for sinus arrhythmias or frequent supraventricular premature contractions. However, in an analysis of 272 consecutive cases of inappropriate diagnosis in a cardiovascular outpatient clinic, atrial fibrillation were more frequently overlooked (51 cases: 18.8%).^17^ It is possible that relatively large f‐waves were misidentified for P wave, resulting in a misdiagnosis as first degree or second degree AV block, sinus arrhythmia, or supraventricular premature contractions, etc (Figure 5).

## 5 FACTORS THAT MAY LEAD TO INAPPROPRIATE DIAGNOSIS

Inappropriate ECG diagnosis can be caused by poor recognition of waveforms as well as several other factors.

Table 2 shows the main possible factors. A combination of multiple factors may lead to inappropriate diagnosis in most cases.

In particular, “recognition of P waves” was a primary factor greatly affecting diagnostic accuracy. The P wave is small, lenient, and sometimes flat. Furthermore, it may become negative or sharpened depending on various conditions.

Accurate recognition of P waves is sometimes difficult even for experts and automatic diagnosis would require a fairly high level of advanced technology. However, in order to further improve the accuracy of automatic ECG diagnosis, it is essential to further improve P wave recognition. Conventional approaches for improving the measurement accuracy of ECG signals have been introduced. Application of AIs with an appropriate method including deep neural network, etc, may result in a breakthrough for further improvements.

It should be noted that studies using AI is progressing in individual applications, such as the evaluation of ischemic heart disease severity or the risk of atrial fibrillation development from the ECG findings.^18^, ^19^, ^20^ However, the utilization of AI in a complete ECG system for automated diagnosis is still in the early stages.^21^, ^22^

## 6 CONCLUSION

Although the accuracy of automated ECG interpretation currently used in Japan and overseas is high, inappropriate diagnosis or ambiguous notations in actual clinical practice is not uncommon. However, there have been very few studies that have clarified the actual situation of inappropriate diagnosis. Inappropriate diagnosis or misdiagnosis leads to unneeded re‐examinations or unnecessary consultations with a specialist. In contrast, adequate and essential treatment may not be received as serious abnormal findings may have been overlooked. Further improvement in the accuracy of automated diagnosis is an urgent need.

## CONFLICT OF INTEREST

All authors declare no conflict of interests to this article.

## MEMBERS OF OUR TASK FORCE (IN NO PARTICULAR ORDER)

### [Core Members]

Masayasu Hiraoka, Takao Katoh, Naohiko Takahashi, Eiichi Watanabe, Takanori Ikeda, Yuji Kasamaki, Naokata Sumitomo, Masaaki Yashima, Norihiro Ueda, Hiroshi Morita.

### [Advisors]

Yoshifusa Aizawa, Hiroshi Inoue, Toru Oe, Noboru Okamoto, Satoshi Ogawa, Ken Okumura, Itsuo Kodama, Kaoru Sugi, Teruhisa Tanabe, Kazunobu Yamauchi.

### [Members]

Takashi Ashihara, Ritsuko Kono, Yoshinori Kobayashi, Tsuyoshi Shiga, Wataru Shimizu, Masaomi Chinushi, Mikiko Nakagawa, Kenji Nakai, Makoto Hirai, Kazutaka Aonuma, Shiro Kamakura, Yoichi Kobayashi, Shingo Sasaki, Ken Kato, Akiko Chishaki, Ayano Minoura, Yuji Murakawa, Akira Yamashina, Shigeyuki Watanabe, Naomi Izumida, Takashi Wada, Yoshiko Furukawa, Daiya Ushinohama, Hitoshi Horigome, Tsuyoshi Yamauchi, Takuya Ishiguro, Takashi Kaiami, Yusuke Kagamihara, Jiro Suto, Eiji Yamaguchi, Tatsuya Yoneyama.
